# An Industry Perspective on Compassionate Use in Europe: A Call for Change

**DOI:** 10.1007/s43441-025-00902-y

**Published:** 2025-12-15

**Authors:** Philipp Schlatter, Nina Heiss, Pedro Franco, Annie O’Keefe Martin, Susan Robson, Ramona Reichenbach

**Affiliations:** 1 F. Hoffmann - La Roche Ltd., Grenzacherstrasse 124, CH-4070 Basel, Switzerland; 2 Merck Healthcare KGaA, Frankfurter Str. 250, 64293 Darmstadt, Germany; 3 Sanofi UK, 410 Thames Valley Park Dr, Earley, Reading, RG6 1PT UK; 4 Novartis Pharma AG, Lichtstrasse 35, CH-4056 Basel, Switzerland

**Keywords:** Compassionate use, Early access pathways, European pharmaceutical legislation, Equitable access, Unauthorized medicines, Regulatory harmonization, Individual patient request, Cohort programme

## Abstract

**Background:**

Options for patients to receive unauthorised medicines through compassionate use (CU) in Europe vary greatly. There are two CU pathways: cohort programmes, regulated uniformly by the Regulation across EU member states, and individual patient requests (IPRs) governed by the Directive. The latter allows member states to determine their own laws, resulting in heterogeneous regulatory requirements and challenges in operationalization. Consequently, patients may experience delays in accessing CU medicines depending on their country of residence. To compare CU availability across European countries and formulate recommendations for improvement, we analyzed 8,934 patient requests from 30 European countries.

**Methods:**

An exploratory post-hoc analysis was conducted using pooled collaborative data from 8,934 patient requests provided by Merck KGaA, Novartis, Roche, and Sanofi, tracked from January 1, 2020 to April 30, 2023 across 30 countries. All requests with complete dates for submission, company approval, relevant Ethics Committees or Health Authorities, and shipment dates were included.

**Results:**

While internal company CU approval steps were found to be similar with a median approval time of 5 days (median interquartile range (IQR) of 1 (0–6) for cohorts; median IQR 4 (1–8) for IPRs), the time from company approval until shipment varied between cohort requests (median IQR 5 (1–16) days) and IPRs (median IQR 8 (1–22) days). Challenges experienced included differences in the use of CU terminologies, scope, settings, regulatory, and operational requirements.

**Conclusion:**

Our findings indicate that differing national requirements across Europe lead to operational challenges and inter-country variability in CU implementation posing operational challenges for stakeholders, highlighting the need for improved harmonization.

## Introduction 

In the European Regulation EC No 726/2004, Article 83, compassionate use (CU) is defined as “‘*compassionate use’ means making a medicinal product available for compassionate reasons to a group of patients with a chronically or seriously debilitating disease or whose disease is considered to be life-threatening, and who cannot be treated satisfactorily by an authorised medicinal *product [1]. The EMA guideline on CU [2], in addition to the Regulation, further clarifies the definition by stating that an unauthorised medicinal product in a CU programme (CUP) is a treatment option for patients who are unable to participate in a clinical trial. Thus, patients with life-threatening or seriously debilitating diseases who have exhausted all commercially available treatment options and are ineligible to participate in a clinical trial may seek access to locally unauthorized medicines through the regulatory pathway known as CU.

There are two types of CU pathways in Europe, one is for CU programmes (also referred to as cohorts) and the other for single or individual patient requests (IPRs). It is important to differentiate between a cohort (program) and an individual patient request (IPR), as they follow two different regulatory pathways at the European and national levels. This distinction matters in practice because while cohort pathways allow for predefined eligibility, IPRs represent individual cases and are thus more variable. Providing CU to cohorts of patients is regulated under the EU Regulation EC No 726/2004, Article 83 [1] while providing CU to IPRs is under the Directive 2001/83/EC, Article 5 [3]. The main difference between the European Regulation and the Directive lies in their binding nature and how they are implemented. The Regulation is a binding legislative act that applies directly and uniformly across all EU member states, becoming part of their national law without needing further national legislation. In contrast, the Directive is binding at the level of the member states and leaves it up to each individual country to decide how to incorporate the directive's provisions into their national law. This legal divergence contributes to varying national implementations and may impact timelines and patient access.

The term “compassionate use” is specifically used by the Regulation [1]. However, in practice, there is no guidance on the use of this term, leading to the use of different and varied terms globally and across Europe by different stakeholders (e.g. early access, managed access) [4]. Some of these terminology variants are listed in the article by the European QP Association IMP Working Group [5]. However, there continues to be confusion, lack of transparency and awareness regarding the meaning and implications of this treatment option.

The Regulation outlines the requirements for CU only at a very top-line level [1], resulting in varying interpretations within the EU members states. Furthermore, because most legislative and operational requirements are under the remit of individual member states which have diverse legislation and policies, if any at all, this leads to varied applicability and complexity in operationalization. Consequently, there is a disparity in the availability of CU across the EU [5].

**Fig. 1:**
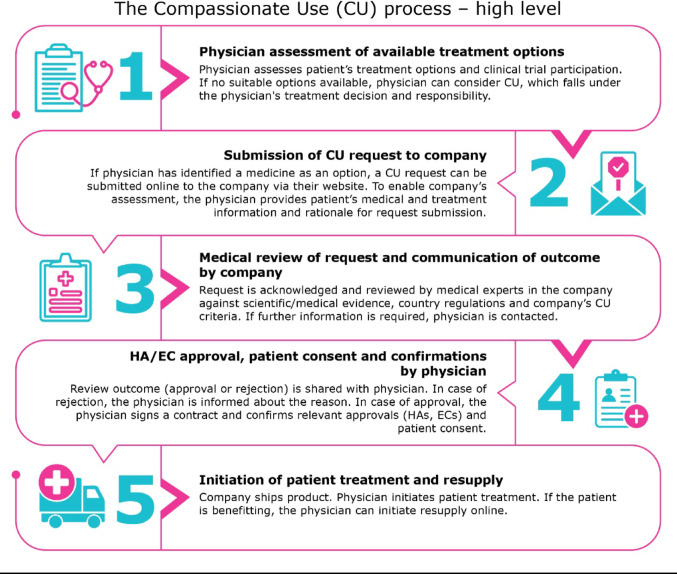
High level overview of the CU process, applicable to all four companies for both cohorts and IPRs. These steps can be iterative and are not necessarily linear.

We would like to emphasize that it is common practice for pharmaceutical companies to adhere to the ethical principle that patients should primarily participate in a clinical trial setting. This is included in the company review criteria and checklist for CU [6]. The request can only be approved if there is no clinical trial available for which the patient is eligible. For these patients, CU may be the only remaining treatment option [7]. While it is up to the company to decide whether to offer CU or not, the CU application is initiated by a health care professional (HCP). Thereafter, the request follows a strict analysis and review by the company, the national regulator and the ethics committee, as applicable (see Fig. 1 for high-level process). The CU request will only be approved if the benefit-risk profile of the product is positive, and if the patient cannot be enrolled in clinical trials or treated with an already commercialized medicine. CU is granted only in situations where no other last resort options are available. To ensure patient safety, the company has an obligation to report serious adverse events to health authorities in accordance with local laws and regulations.

Various stakeholders dealing with CU (health care professionals, pharmacists, nurses and patients) have identified the current challenges from their perspectives. For example, the EU network of qualified persons (QP) [6] and European Organisation for Rare Diseases (EURORDIS), an EU patient organization [8], have proposed strategies to achieve greater harmonization and convergence. The current revision of the European Pharmaceutical Legislation provides a critical opportunity to implement changes to enhance the simplification and harmonization of operationalising CU availability, ultimately to the benefit of patients. Specifically, the European Commission’s Proposal for the revised Regulation of the European Parliament and of the Council (COM(2023) 193 final 2023/0131(COD)) was published on 26th April, 2024 and did not introduce any changes applicable to compassionate use. Negotiations with the European Parliament and the Council were completed and an agreement on the legislative package was reached on December 11th 2025, prior to official publication in the EU's Official Journal in 2026. . One objective of this manuscript is to continue to educate on the changes that are needed to improve on the new Article 26 of the new Regulation, which replaces Article 83 of the previous Regulation [9].

We acknowledge that harmonization alone will not guarantee equitable and rapid access, as several other factors also play a significant role e.g. awareness on CU options, a manufacturer’s ability or willingness to supply the medicine, and the varying health system infrastructures across Europe. Nonetheless, streamlining the legislative pathways would be a crucial step toward improving access to CU.

To demonstrate the importance of streamlining and enhancing transparency on CU for all stakeholders involved including HCPs and patients, four major pharmaceutical companies (Merck, Novartis, Roche, Sanofi) worked collectively to pool cross-company data of 8,934 patient requests. This data offers broad coverage across 30 European countries to analyse and share the challenges associated with operationalising CU in Europe. The analysis aimed to highlight patterns of CU access. We examine and discuss the variability in access to CU in Europe from an industry perspective. Further, based on our analysis and wider community experience, we propose recommendations for further convergence, harmonisation and transparency to accelerate the availability of innovative medicines through CU for seriously ill patients across Europe.^1^

## Materials and Methods

The data analyzed was derived from data points routinely tracked for CU requests within each pharmaceutical company’s system (Merck, Novartis, Roche, Sanofi) during the timeframe from January 1, 2020, to April 30, 2023. The analysis included data from the following 30 European countries: Austria, Belgium, Bulgaria, Croatia, Cyprus, Czech Republic, Denmark, Estonia, Finland, France, Germany, Greece, Hungary, Iceland, Ireland, Italy, Latvia, Lithuania, Luxemburg, Malta, Netherlands, Norway, Poland, Portugal, Romania, Sweden, Slovakia, Slovenia, Spain and the United Kingdom. While not all four companies contributed requests from every country, the pooled data collectively covered 30 countries. This is because CU requests are always unsolicited, and it is not the companies who determine in which countries to provide CU.

Requests from the two types of CU regulatory pathways (cohorts and IPRs) were included in the analysis. As a principle, only first patient requests that we recorded in the data set for a specific patient was included in the analysis. We did not consider second requests for re-supply as they were not received for all patients. For a CU cohort, the eligibility criteria were pre-defined by the company and aligned with the anticipated regulatory submission for market approval. The CU protocol was submitted at the national level and the approval pathway varies by the country. For IPRs, the research data is often inherently at a very early stage and the eligibility check is based on early scientific evidence. Thus, the inclusion and approval criteria in these cases are less predefined and handled on a case-by-case basis. In this study, only those requests that were approved by the companies were analysed. All patient requests were approved by the companies, Ethics Committees and Health Authorities as applicable according to local regulations. Patient consent was obtained by the physician in accordance with local laws and regulations.

All patient identifiers were removed to ensure confidentiality. The following information was tracked in an Excel sheet: country of request, type of request, date of submission by physician, date of company approval, date of medicine shipment. Requests with the dates in sequence were considered to fulfill the completeness requirements and thus considered in the analysis. The data set was cleaned to remove erroneous or partial information. As mentioned earlier, only initial requests made within the timeframe of January 1, 2020 to April 30, 2023 were included. All requests representing second patient requests, where the initial request did not fall within the defined cut-off dates were excluded.The requests were received in real-time and handled as they came in due to the unsolicited nature of HCPs submitting CU requests. The data was simply tracked and reported. All requests that fulfilled the criteria of a CU case were included. Only requests with all completed dates, including shipment dates, were part of the analysis.

The data was pooled for the analysis and examined by country and request type (cohort vs IPR). Simple descriptive statistics and graphs were used. We did not perform an inter-company analysis or stratification because the objective was to holistically analyse the entire data set and not to compare company outputs.

The following data points were used for analysis: country of request, type of request (program or IPR), dummy product ID number, date of submission of the request by the physician, the date of company approval, and the date of medicine shipment. The number of days that we refer to throughout this manuscript, represent two values: first, the calendar days between the date of submission and the date of company approval, and second, the days from company approval to the date of medicine shipment. The data was maintained in an Excel sheet for filtering, sorting, and determining the number of calendar days.

This was an exploratory post-hoc analysis based on collaborative data provided, analysed, and reviewed by the companies (not all companies were covered for IPR). We described the current situation as it was observed. As this was a non-controlled study, a statistical analysis was not performed.

The pooled data represent a snapshot of CU activity in Europe during the allocated time-period. We thus acknowledge the limited generalizability due to potential bias that could not be controlled due to using observational data from four different reporting systems that were retrospectively analysed.

## Results

A total of 8,934 CU requests were received for drugs in clinical development by the four pharmaceutical companies during the time-period from January 1, 2020 with a cut-off date of April 30, 2023.

As mentioned in the introduction, according to the European Pharmaceutical Legislation a differentiation is made between a CU request for a group or cohort of patients through a programme (as by the Regulation) and an IPR (a *bona fide* unsolicited request as by the Directive). Of the total CU requests received, 5,799 were for cohorts, while 3,135 were IPRs. 

In Fig. 1, for illustrative reasons we provide a high-level, simple, descriptive overview of a CU request journey from the initial assessment by the physician until the patient can initiate treatment. These steps were applicable to all four companies involved, they can be iterative and are not necessarily linear. Regulatory complexity for the company occurs between steps 2 and 5 (see also Table 1 for explanations). The physicians are responsible for seeking approval by the national Health Authority (HA), the national Ethics Committees, and seeking patient consent (step 4) in line with local laws/regulations.

**Table 1:** Key challenges experienced by the four companies with a description on the impact on patients and suggested opportunities for improvement from an industry perspective

*Challenge 1: Individual versus cohort access*	
Description	*Article 83 of Regulation (EC) No 726/2004 *governs CU of unauthorized medication to a group of patients under specific criteria e.g. life-threatening conditions. *Article *5 (1) of* Directive 2001/83/EC* deliberately allows member states to provide access to unlicensed medicines to individual patients with different access criteria than Article 83. CU for individual patients as a result falls under the remit of national jurisdictions which create different policies and national requirements in each member state leading to an overly complex path towards providing CU to a patient. Due to this regulatory design, there is no common legislative home in the EU legislation for both CU cohorts and for IPRs.
Impact on patients	CU access across member states is compromised due to different local interpretations of EU Regulation (EC) No. 726/2004 and Article 5 (1) of Directive 2001/83/EC. Patients’ CU options depend on where they live in the EU. Patients in some member states benefit from well-defined cohort and IPR options whereas some member states lack to regulate IPR availability leading to variability in access to CU across the EU.
Recommendation	Revision of the Regulation has led to Article 83 being called Article 26. We recommend to address both a CU cohort and an individual patient request under a unified legal basis to enhance harmonization of regulatory requirements by amending the Article 26 of the revised Regulation to include cohorts and IPRs. We also recommend to update the European guideline on CU.
*Challenge 2: Nomeclature/Scope*	
Description	The EMA Regulation uses the term Compassionate Use in the Regulation. Due to the national complexities, a spectrum of terminologies is used across member states e.g. CU, expanded access, managed access, pre-approval access, early access, named patient supply, extended access, patient access, timely access. These terms are not formally defined by law.
Impact on patients	Confusion across healthcare professionals, patients and industry. Different terms result in delays while companies seek to understand the local regulations and requirements and align these with their internal policy to assign the most appropriate pathway.
Recommendation	We propose to clarify the use of terms by revising the CU guideline. Propose to add the requirement for an update of the CU guideline in the revised Regulation accordingly.
*Challenge 3: Regulatory requirements (including data collection)*	
Description	There are different national requirements set by the individual countries, differing timelines for approvals of CU, and no consistent European framework exists for quality data collection. There are no consistent European QP release requirements for CU, increasing the hurdle to release CU medicine across the EU. QP release needs to comply with national requirements available in local language. No consistent European labelling requirements. Labels must usually be in local language to comply with national requirements. In some member states there is a flexibility in accepting labelling in English for medicines to be administered in a hospital setting. Data collection: Article 83 of the Regulation does not mention the concept of data collection leaving this to the member states. Thus, the type and quality of data collected varies broadly reducing the benefits of data collection. There are cases where CU data was used to support regulatory decision-making for drug approvals demonstrating the value of such data (e.g the FDA approval of a new pellet formulation in the paediatric indication for entrectinib is an example which shows that if the data had not been collected in the context of CU this would have been a missed opportunity).
Impact on patients	Clarity on CU requirements depend on the national legislation/policies/guidelines which leads to inequalities across the EU and impacts patient`s time to CU availability. Patients might benefit from (earlier) availability to treatment if RWD from CU cases would be captured consistently.
Recommendation	We propose to lay down regulatory requirements for structured data collection, label and product information, importation, QP release, expectation on documentation for national assessment, safety reporting, and informed consent. Providing more clarity on data collection on CU will create an opportunity to use this data for meaningful discussions with Health Authorities and Payers.
*Challenge 4: Free of charge vs charged for*	
Description	Often a medicine is provided at no cost until approved or available in the member states. Across the EU, there are member states which: - do not allow medicine given to a patient for free, but a minimal, purely symbolic price must be assigned - do not allow charging at all - have a local CU reimbursement scheme in place
Impact on patients	Patients will experience differences with respect to free of charge versus a charge for receiving a medicine via CU across the EU depending on where they live. This may impact the opportunity or delay availability of treatment. Discrepancies exist in reimbursement of CU from member state to member state.
Recommendation	Learning from other national models in EU (e.g. France ATU/Accès précoce model) and implement best practices.

CU, compassionate use (here refers to CU programmes and Individual Patient Requests); EU, European Union; QP, Qualified Person; RWD, real world data.

**Fig. 2:**
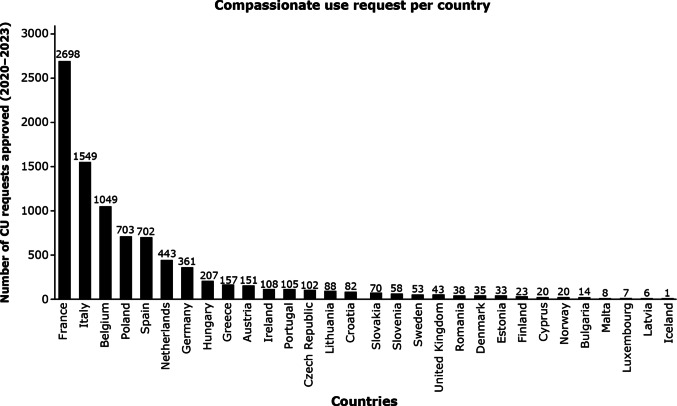
Compassionate Use (CU) requests approved by Merck, Novartis, Roche, and Sanofi in the 27 EU member states plus Iceland, Norway and the UK. Data points (requests) were tracked between January 1, 2020–April 30, 2023

Figure 2 depicts the distribution of the number of CU requests across Europe received within the 3 years and 4 months by the four companies. Requests for CU were received from all 27 EU member states plus Iceland, Norway and the UK. The highest number of approved requests analysed in this analysis were received from France (n = 2698), Italy (n = 1549), Belgium (n = 1049), Poland (n = 703), Spain (n = 702), the Netherlands (n = 443) and Germany (n = 361). Few requests were received and approved for Malta, Luxembourg, Latvia, and Iceland. The variability in the number of requests can be attributed to several factors: the variability in the availability of an enabling national framework for CU, the awareness of the HCP, and the CU case itself.

**Fig. 3:**
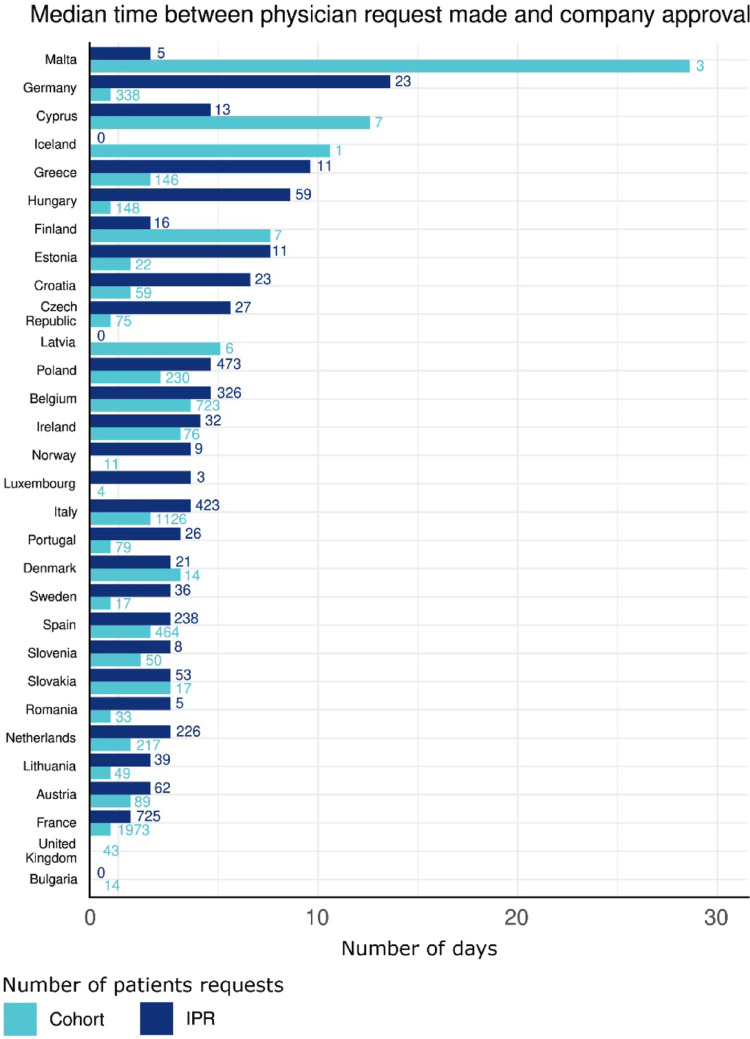
Median time in (days) between date of submission of the request by the physician and the date of company approval for Individual Patient Request in blue (IPR) versus cohort requests. On the X-axis, the median number of calendar days between the physician’s date of submitting the request and the date when company approval was issued is shown separately for IPR and cohort requests. The numbers on the bar depict the number of approved requests in the respective type and country. Missing bars are either “0” for no requests received for that type or when company approval was granted within the same day.

The median time required in days between the date when the request was submitted by the physician until the date of company approval is shown in Fig. 3. Here we compared IPRs against patients allocated to cohorts. Most of the company approvals were granted swiftly within a median time of around 5 days, with a tendency for longer approval timelines for IPR than for cohort requests (median interquartile range (IQR) of 1 (0–6) for cohorts; median IQR 4 (1–8) for IPRs). In some cases, approvals were granted on the same day. This explains the “missing” bars on the graph in some cases because the median time was “zero” days. Another reason for a missing bar is a count of “0” for no requests received for that type.

**Fig. 4:**
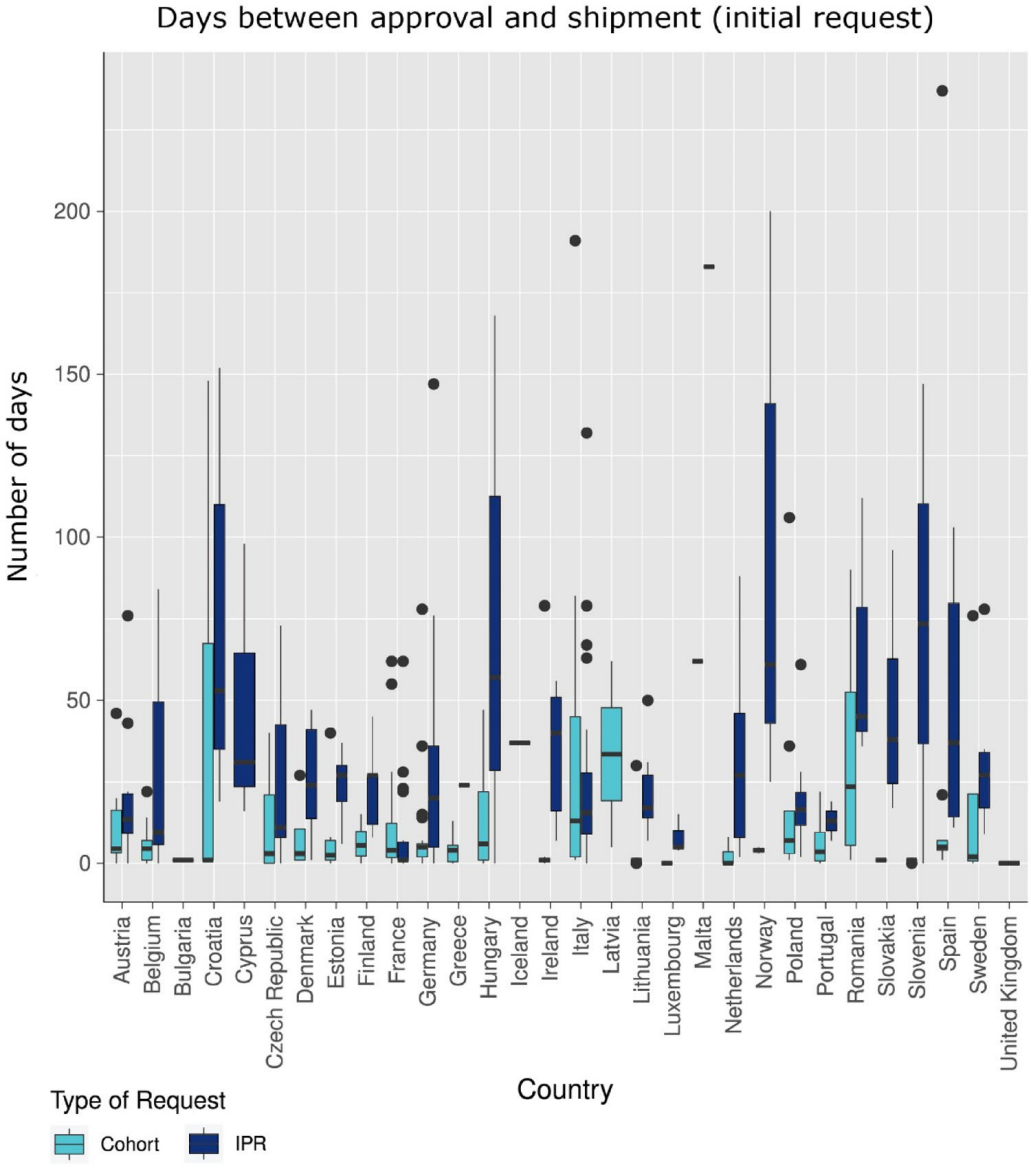
Median time (days) from date of company approval to date of medicine shipment per country showing differences between individual patient requests (IPR) (dark blue) and cohort requests (light blue). This figure shows the raw data collected as it was observed in real-time. The outliers in this plot may be due to incorrectly entered data in the system and should thus not be overinterpreted as the data points (dates) were entered into the system manually.

Figure 4 displays the boxplot of the median days between date of company approval and date of medicine shipment for IPRs in comparison to cohort requests. The median time from approval to shipment, for cohort requests, are significantly shorter (< 10 days). For IPRs higher variances of up to 50 days were seen (cohort (median interquartile range 5 (1–16) days) and individual patient requests (IPR) (median interquartile range 8 (1–22) days)). Note: for countries with less than 3 requests received the median shown should be interpreted with caution especially in cases of outliers as seen in Fig. 4. We acknowledge that some of the durations observed are high. This is due to the nature of this research (post-hoc analysis of routinely collected annynomised real-time data). Only a limited number of requests experienced issues due to system migration errors, resulting in the original shipment date not being populated. In these instances, the system automatically assigned a date based on a predefined rule.

## Discussion

In this article, we provide a descriptive analysis to illustrate the challenges of variability in operationalising CU in Europe from an industry perspective. We recommend areas for improvement to the European legislation that could enhance the availability of CU through more equitable means across Europe.

Despite the challenges, the across-companies dataset indicates that there is a substantial degree of CU activity occurring across all countries. As a result, many patients benefit from this treatment option of receiving an innovative medicine in a pre-approval setting in a clinically supervised environment which includes mandatory safety monitoring and collection of safety data for both cohorts and IPRs. 

However, disparities exist in the likelihood of patients receiving prompt access to unauthorized medicines through CU. Our data aligns with previous assumptions that this depends on the country in which the patient lives in Europe. Company approval times of IPRs typically take longer than for cohort programs which we attribute to the different case-by-case and unique clinical settings of IPRs. In the case of IPRs there are no pre-defined eligibility criteria which makes it more difficult to operationalize the request. This is compounded by the frequent need to obtain additional information from the physician before a final case-by-case decision can be made by the company.

The time from company approval until shipment is longer than the time taken from receiving the request to company approval. This is particularly the case for IPRs where there is also a higher degree of variance. This is because after company approval there are external factors such as different regulatory and operational requirements in each country, which the company cannot influence. The external factors that the company can not solely control include complex operational activities such as executing a contract with the physician, often requiring local legal negotiations, obtaining Ethics Committee/Health Authority approvals, and receiving required test results (such as genotype testing). Further, the documentation required for Ethics Committee/ Health Authority approvals vary often leading to extensive paperwork and extended completion timelines. Furthermore, the arrangement of medicine supplies is complex because they differ at a local level (e.g. labelling, Quality Person (QP, an expert legally responsible for certifying each batch of a medicine released from a manufacturing facility within the EU), release and importation). In summary, streamlined navigation in execution is hindered by unharmonized regulatory requirements across Europe for these multiple steps. 

Although our analysis of observed data represents a large data set, the study does come with limitations and potential bias. Firstly, we represent the company perspective only and not a comprehensive view across all stakeholders. Secondly, the sample pool is limited in that it does not represent all CU activity in Europe during that time-frame. Smaller companies, in particular, may face different challenges, which could introduce bias, as our findings primarily represent the views of medium to large-sized pharmaceutical companies. Thirdly, this is a purely observational data. We did not perform any statistical testing and stratification.

While median timelines were generally short (< 5 days), considerable inter-country variation was observed. This, together with our experience supports the assumption that the inherent variability in lead times that influence CU availability in Europe is due to the differences in European legislation. The Regulation Article 83 (Regulation (EC) No 726/2004 [1] was introduced in 2004 enabling CU to only groups of patients (cohort programmes) and not covering IPRs. The requirements outlined are very high-level. In essence, managing a CU request remains under the remit of the individual jurisdictions that exist in the various member states. Each member state has its own local policies and national requirements for operationalising CU and divergent national clinical practices leading to inconsistency across Europe. In countries with well-established more enabling CU frameworks there tends to also be a higher awareness of the existence of CU as a potentially life-saving option. For example, France has a well-established CU framework and our study demonstrates that France had the highest number of requests and the shortest time-lines for both cohorts and IPRs.

An even higher degree of variability is seen for IPRs. Article 5 of Directive 2001/83/EC [3] provides a legal basis to a member state to allow the supply of unauthorised medicines to individual patients (single patient, named patient request), but the operationalisation remains with each member state based on local regulatory requirements. The different approaches with regard to cohort versus IPR access increases the regulatory complexity leading to delays. For example, in Germany, the national legislation covers only CU for cohorts. These are called “Hardship Cases” in which the German health authority is involved. However, in cases described as a “compelling medical need”, the IPR is covered by the criminal law (captured in § 34 of the Strafgesetzbuch (StGB); rechtfertigender Notstand). The justification to provide CU is an individual exceptional case based on a concrete emergency.

Conversely, in France, both IPRs (Accès Compassionnel) and cohorts (Autorisation d’accès précoce) are covered by the same legislation. 

Based on our experience, and as reported in detail previously, France, Belgium, Italy and the Netherlands have a robust and mature scheme for CU programmes [5]. France, for example, was the first country with a CU national legislation in Europe in 1994 with an elaborate scheme for CUP called Temporary Authorizations for Use (ATU) [5]. We attribute the maturity level to the higher level of activity seen in these countries. Despite these comprehensive schemes, we still encounter differences. This we explain by the variable understanding and awareness around the topic of CU among HCPs and in the healthcare community as a whole.

Countries like Malta, Cyprus, Latvia, and Luxembourg had low request volumes, which may be due to a lower awareness on the option of CU in these countries. There is an ethical concern of unequal access based on geography. Europe has the opportunity to strengthen implementation of the new regulation.

In summary, countries with the higher number of requests have better CU frameworks, and physicians in these regions have higher awareness on the CU option. However, due to the complexity of the CU process, and the fact that physicians are dealing with critical cases on a case-by-case basis, it is not exclusively possible to say which single factor or factors drove the highest numbers in each instance.

In Table 1 we summarize the key challenges that industry faces when providing a CU medicine to a patient in Europe, in conjunction with proposed recommendations for improvement and amendments to the legislation. To complement Table 1, we would like to specifically call out three key challenges and highlight examples from selected countries to illustrate the points. This section is followed by a discussion on the recommendations.

### Labelling Requirements

Another example of complexity arises from labelling requirements. In France, special labels are required for the cohorts whereas in Germany, labels are required to include an additional statement indicating “for CU only”. The requirement for labels to be in the local language, as mandated by some countries, can also lead to delays. However, in some countries like Romania, labels can be provided in the local language, English, or French. Especially when dealing with urgent IPRs, the need to create and produce a separate label can cause substantial delays. This time is lost for seriously ill patients who may not have that length of time to wait for their treatment. The operational steps mentioned above could be simplified and clarified by including harmonized requirements in an updated guideline on CU.

### QP Requirements

The requirements for the release of a medicine for CU by a Qualified Person (QP) are not harmonised within the EU, making it difficult for companies to accurately navigate the legislation on a country-by-country basis. In most EU countries the importation of a medicine necessitates a QP release in one EU country, however there are exceptions to this rule [6].

### Real World Data (RWD) Collection

The topic of data collection is also a challenging component when dealing with CU cases. Data collection is not the primary intent of CU, but it can further enhance the evidence base of the medicine used in the patient population, especially in context of rare diseases [10]. While we recognize that RWD comes with challenges such as bias and lack of comparators, it is increasingly being used to support evidence packages for approval. RWD from CU has been used as a source of data to support FDA [11] and EMA [12] approvals. RWD from CU programs is increasingly valued by the Health Authorities, HTA (Health Technology Assessment) bodies, payers, industry, and patients as it complements data from clinical trials in the real-world situation. However, for CU cases, EU countries have different positions on data collection: France mandates it by law, Italy has an optional provision for some clinical data collection, while Germany lacks explicit regulations, with decisions often depending on the specific program. As a result, the extent, quality, and types of data collected are heterogeneous, limiting their utility in contributing to sources of RWE. We propose to include guidance on a harmonised framework for data collection in the updated CU guideline (e.g., demographics, disease characteristics, treatment exposure, key outcomes) and provisions for GDPR-compliant consent forms harmonized across the EU [2].

Based on our experience, patients are unaware of the CU setting and physicians often only learn about this option once they conduct clinical research and are exposed to the pre-approval environment. This lack of awareness generally hinders patients with unmet medical needs from accessing early and innovative treatments.

Overall, the complexities, lack of transparency, awareness, and education regarding CU highlight significant areas for improvement such as streamlined European regulations.

Aliu et al. [13] reported that countries with robust CU legislative frameworks enables CU activity, leading to less variability in access. They describe an 8-factor robustness framework for CU regulations that could enhance national legislations. However, we continue to observe that the current regulatory framework does not provide sufficient clarity, transparency and equality in the provision of CU across Europe.

As part of the implementation of the revised General Pharmaceutical Legislation, we would like to present a few recommendations for consideration to improve the legislation at the European level. Further elaborating on what is described in Table 1, we are taking the position that on top of the minor changes proposed by the European Commission [14], the proposals made by the European Parliament to amend the Regulation [15], could make a crucial contribution to improve the CU situation in Europe. Firstly, as by the position of the European Parliament, we propose to address both cohorts and IPRs in the Regulation.

A second change that we recommend is to require that the Agency make CU notifications publicly available. We suggest introducing a publicly and centrally available European CU register, which would enhance visibility and awareness for all stakeholders involved.

Furthermore, because the European Guideline on Compassionate Use [2] does not provide explicit guidance on how to operationalize a CU request within the current legal framework, we propose to update the guideline by including harmonized and more detailed guidance on the steps that lead to delays (e.g. labelling, importation, QP release see Table 1 for details).

Another step forward would be to enable a more consistent and structured collection of RWD across Europe. A documented permission for the use of this RWD could be achieved via the associated informed consent process. The EU Parliament has proposed an important amendment to Article 26, stating that “the Committee may also make use of health data generated outside of clinical studies, including RWD”. This change should be accompanied by an update in the existing guideline [2] (or the introduction of additional guidelines) to provide a structured framework for the types of RWD to be systematically collected to enhance overall quality and support regulatory filings.

The identified challenges create a need for harmonization across Europe, potentially implementable with only minor changes in the legislation in order to avoid delays for patients with a high unmedical need to access an unauthorized medicine through CU. The potential impact of not receiving a treatment in time, or during a window of opportunity for maximum benefit, can be devastating for a patient’s life and even lead to death.

## Conclusion

CU is a treatment option for some severely ill patients. There is an ethical responsibility to facilitate CU availability in a timely and equitable manner that does not favour one patient over the other solely due to the geographic location. Our analysis is in line with the assumption and our experience that inequalities exist in Europe for patients to receive a medicine in a timely way through CU as this depends on the country in which the patient lives. It would be of great value to patients if some of the main barriers of not having single patients and cohorts embedded in one European Regulation and stakeholders having to deal with the many complex and heterogeneous national legislations were addressed as by our recommendations. More harmonization that leaves flexibility where access is not impeded would balance CU access with the necessary regulatory oversight and operational feasibility. We urge policy-makers and regulators to pay more attention to the hurdles that stakeholders face when addressing a CU request. Implementation of the revised pharmaceutical legislation by the European Commission, the European Parliament, and the Council continues to represent a unique opportunity to engage in this discussion in the interest of patients who are most in need.

## Data Availability

No datasets were generated or analysed during the current study.

## Footnotes

1 CU, compassionate use; HA, Health authority; EC, Ethics committee.
